# Transcriptome analysis of microRNAs in developing cerebral cortex of rat

**DOI:** 10.1186/1471-2164-13-232

**Published:** 2012-06-12

**Authors:** Mao-jin Yao, Gang Chen, Ping-ping Zhao, Ming-hua Lu, Jiang Jian, Mo-fang Liu, Xiao-bing Yuan

**Affiliations:** 1 Institute of Neuroscience and State Key Laboratory of Neuroscience, Institutes for Biological Sciences, Chinese Academy of Sciences, Shanghai, 200031, China; 2 Graduate School of the Chinese Academy of Sciences, Shanghai, 200031, China; 3 State Key Laboratory of Molecular Biology, Institute of Biochemistry and Cell Biology, Chinese Academy of Sciences, Shanghai, 200031, China; 4 Shanghai Institutes for Biological Sciences, Chinese Academy of Sciences, Shanghai, 200031, China

**Keywords:** MicroRNA, RNA editing, Cerebral cortex, Development

## Abstract

**Background:**

The morphogenesis of the cerebral cortex depends on the precise control of gene expression during development. Small non-coding RNAs, including microRNAs and other groups of small RNAs, play profound roles in various physiological and pathological processes via their regulation of gene expression. A systematic analysis of the expression profile of small non-coding RNAs in developing cortical tissues is important for clarifying the gene regulation networks mediating key developmental events during cortical morphogenesis.

**Results:**

Global profiling of the small RNA transcriptome was carried out in rat cerebral cortex from E10 till P28 using next-generation sequencing technique. We found an extraordinary degree of developmental stage-specific expression of a large group of microRNAs. A group of novel microRNAs with functional hints were identified, and brain-enriched expression and Dicer-dependent production of high-abundant novel microRNAs were validated. Profound editing of known microRNAs at “seed” sequence and flanking sequence was observed, with much higher editing events detected at late postnatal stages than embryonic stages, suggesting the necessity of microRNA editing for the fine tuning of gene expression during the formation of complicated synaptic connections at postnatal stages.

**Conclusion:**

Our analysis reveals extensive regulation of microRNAs during cortical development. The dataset described here will be a valuable resource for clarifying new regulatory mechanisms for cortical development and diseases and will greatly contribute to our understanding of the divergence, modification, and function of microRNAs.

## Background

The mammalian cerebral cortex contains a large number of neurons of different phenotypes arranging in a stereotypical laminar pattern [1]. A series of sequential cellular events happen during cortical development, including neural progenitor proliferation, cell fate specification, neuronal migration, neurite outgrowth and pathfinding, and eventually the formation and plastic modulation of synaptic connections [2]. The happening of all these developmental events depends on the precise spatial and temporal control of gene expression in the cell. Extensive studies have been carried out to clarify the role of transcription factors, including activators and repressors, in the regulation of gene transcription during these developmental events. In addition to transcriptional regulation, various types of small non-coding RNAs in the cell have been shown to play significant roles in the control of gene expression during physiological and pathological processes [3], largely increasing the complexity and flexibility of the gene regulatory network. MicroRNAs (miRNAs) are a group of most extensively studied small RNAs of around 18–24 nucleotide (nt) with the typical stem-loop structure [4]. Most mature miRNAs directly interact with a group of messenger RNAs (mRNAs) and suppress their expression either by guiding the cleavage of the target mRNAs or by inhibiting their translation upon imperfect base pairing to mRNA’s 3′- untranslated region (3′-UTR) [4]. Interestingly, some mature miRNAs can undergo changes of one or more nucleotides in their “seed” sequence, a process known as miRNA editing, which further increases the complexity of gene regulation [5]. In addition to miRNAs, other classes of small RNAs, including repeat associated small interference RNA (rasiRNA), PIWI-interacting RNA (piRNA), and small RNAs derived from transfer RNA (tRNA), ribosomal RNA (rRNA), small nucleolar RNA (snoRNA), small nuclear ribonucleic acid RNA (snRNA), small cytoplasmic RNA (scRNA), and signal recognition particle RNA (srpRNA), also play constitutive or regulatory functions in various cellular events.

A number of brain miRNAs appear to be developmentally regulated, with high expression in neural progenitors but not in differentiated neurons, or vice versa [6], suggesting that they may function at different stages of neuronal development [7]. As well characterized examples, miR-9 has been shown to regulate embryonic neurogenesis by targeting the transcription factor TLX [8]; miR-219 [9] and miR-338 [10] have been identified as regulators of oligodendrocyte differentiation; miR-124 have been shown to promote neuronal differentiation and regulate adult neurogenesis [11,12]; and miR-134 have been shown to regulate dendritic spine morphology through inhibiting the local translation of Limk1 [13]. Links between miRNA dysfunction and neurological diseases have become more and more apparent. For example, mutation in the seed region of miR-184 causes familial keratoconus with cataract [14] and mutations in the seed region of miR-96 are responsible for nonsyndromic progressive hearing loss [15]. Variation in the miR-433 binding site of FGF20 confers risk for Parkinson diseases by up-regulation of α-Synucein [16]. Interference of miRNA biogenesis by disrupting the miRNA processing enzyme Dicer in the nervous system has provided the evidences that miRNAs are essential for the development of the nervous system [17-20]. Conditional knock-out of Dicer in the mouse telencephalon resulted in a size reduction of the forebrain, likely caused by apoptosis of differentiating neurons [20]. Similar neuronal death was observed when Dicer was inactivated postnatally in the cerebellum [17] or in dopaminergic neurons in the midbrain [19]. These findings are consistent with an important role of miRNAs in regulation of cell proliferation, survival, and differentiation in developing brain. However, which miRNAs are expressed at different developmental stages and how various miRNAs are engaged in the regulation of each developmental event remain largely unknown.

Recently, next-generation sequencing has emerged as a powerful tool for clarifying the expression profile of small RNAs. The advantages of the massive parallel sequencing technique lie in its unbiased high-throughput detection of small RNAs at a genome-wide scale, even for low-abundance transcripts, and in its unparalleled ability in identifying novel RNA transcripts and modification of RNAs such as RNA editing. Although the next-generation sequencing had started to be used to examine the brain transcriptome [21], a systematic analysis of miRNAs in developing brain using this new high-throughput method is largely lacking.

In the present study, we applied the next-generation sequencing technique to carry out a systematic analysis of miRNAs isolated from rat neocortex of many developmental stages. In addition to the demonstration of dynamic and stage-specific expression of a large group of known miRNAs, we identified a group of novel miRNA candidates in rat cortex with functional hints. Interestingly, we observed profound nucleotide editing of “seed” and flanking sequences of miRNAs during cortical development. The dataset described here will be a valuable resource for clarifying new regulatory mechanisms for cortical development and disease and will greatly contribute to our understanding of the divergence, modification, and function of miRNAs.

## Results

### 

**Figure 1:**
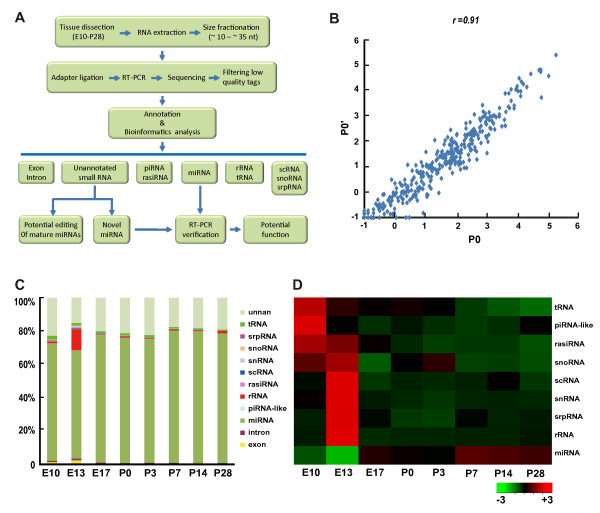
**Overview of deep-sequence results.A.** Flow chart of the study. Briefly, RNAs were extracted from rat cortical tissues of different developmental stages, size selected, and then sequenced by using the Solexa 1 G genome analyzer. Clean read tags were annotated by different bioinformatics softwares (see methods). **B.** The comparison of read numbers per miRNA between the two P0 samples. Since the read number per miRNA ranges from 0 to >10,000, the read number adding 1 was transformed by log10. Each dot represents data from one miRNA. There is a high correlation between the two sequencing results (*r* = 0.91, Pearson’s correlation; p < 0.001(two tail). **C-D.** Relative abundances of different classes of small RNAs. The chart (**C**) show the relative levels of each of the nine classes of small RNAs at different developmental stages. There were <3% reads coming from the degradation of mRNAs (exon and intron). The heat-map (**D**) shows the developmental tendency of the total amount of each class of small RNAs. Red and green indicate high and low expression, respectively.

As shown in the work flow (Figure 1A), RNA samples were extracted from rat cortical tissues of eight developmental stages (E10-P28). A RNA integrity number (RIN) was evaluated to monitor the general quality of extracted RNA samples [22]. As shown in Figure  S1, RIN of all samples are ≥8.4, indicating high quality and low degradation of these samples [22]. RNA samples were size-selected (10–30 nt) and sequenced by Solexa technique [23]. Two independent P0 samples were assayed (P0 and P0’, biological replicates) in order to evaluate the reproducibility of the experimental procedures. Each sample was sequenced twice (technical replicates) and results were averaged to reduce experimental errors. We obtained approximately 20 million total reads for each sample after removal of low quality reads and contaminants (Table S1, see methods), with the peak length of each sample at about 20–22 nt (Figure S2). Small RNA reads >18 nt were annotated based on their sequences, and their relative abundances were determined by their counts, normalized to the total read number and shown as transcripts per million reads (TPM, see methods) [24]. To minimize the false positive signal, only reads that were detected in both two sequencings (technical replicates) were used for further bioinformatics analysis.

This small RNA quantification based on deep-sequencing was highly reproducible, as reflected by a high Pearson’s correlation coefficient between miRNA levels of the two independent P0 tissue samples (r = 0.91) (Figure 1B). Consistent with a peak of the length distribution at around 20–22 nt, we found that miRNAs were the major fraction of small RNAs detected in rat cortex at all developmental stages (≈70%, Figure 1C). rRNAs are known to play important roles in the protein synthesis machinery. Interestingly, small RNAs derived from rRNA at E13 were significantly higher than all other stages (Figure 1C-D). Consistently, as shown in Figure 1D, the total expression levels for small RNAs derived from scRNAs, snRNAs, and snoRNAs, three groups of small RNAs that contribute to the biogenesis of rRNAs or to the protein synthesis, all significantly correlated with that of rRNA-derived small RNAs, with a peak at E13. Since E13 is characterized by onset of neurogenesis in rat cerebral cortex [25], the peak of rRNA-derived small RNAs at E13 suggests an important role of regulation of protein synthesis for the onset of cortical neurogenesis. Other classes of small RNAs detected in cortical tissues, including piRNA-like RNAs and rasiRNAs as well as those derived from tRNAs and srpRNAs, exhibited gradual reduction in their expression during development.

### Identifying and profiling of known miRNAs

**Figure 2:**
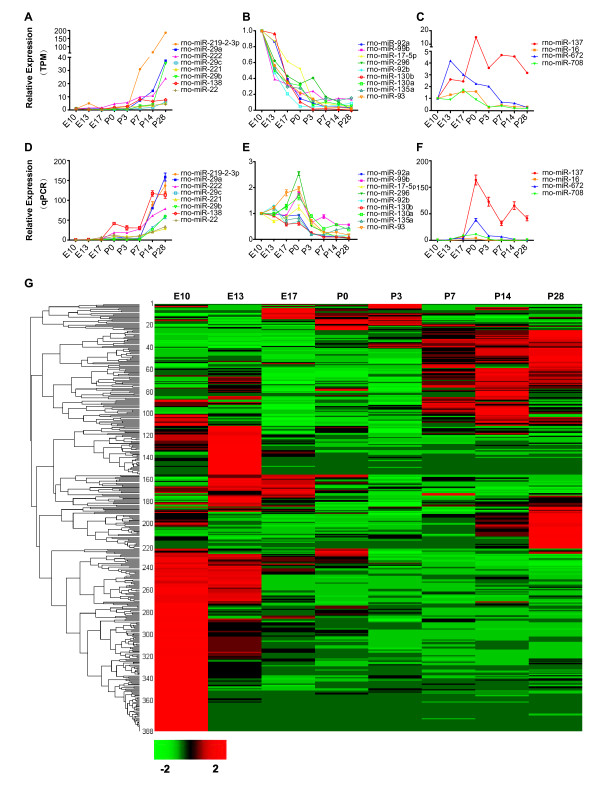
**Validation and clustering analysis of developmentally regulated miRNAs.A-C**. The expression of three groups of miRNAs at different developmental stages revealed by deep-sequencing. The three groups are: gradual increasing (**A**), gradual decreasing (**B**), and peak at middle stages (**C**). The expression level of miRNAs (TPM) at each developmental stage was normalized to that at E10. **D-F.** Quantitative PCR (qPCR) analysis of the expression profile of different groups of miRNAs shown in **A-C.** Results are based on average of three independent experiments (Mean ± SD). The developmental changes of the expression of most miRNAs revealed by qPCR are consistent with results from deep-sequencing. **G.** Clustering of differentially expressed microRNAs. The Complete Linkage Clustering was used by R package based on expression levels (TPM) of each miRNA at different stages. Both known miRNAs and novel miRNAs are included (Dataset S1). Red means highly expressed and green means lowly expressed.

By aligning clean reads to precursors of known miRNAs in the miRBase (release 18.0) [26], we identified approximately 280 known miRNAs and 55 miRNA* (miRNAs star) expressed in cortical tissues of at least one of the eight developmental stages (Table 1). Currently, there are 438 mature rno-miRNAs and 242 rno-miRNAs* deposited in miRBase database, and close to fifty percent of these known miRNAs are expressed in rat cortex. To further validate the deep-sequencing results, we chose 21 miRNAs with typical expression profile during development (gradual increasing, gradual decreasing, and peak around P0) for further analysis using the quantitative polymerase chain reaction (qPCR) [27]. We found that the expression patterns of most of these miRNAs revealed by qPCR were consistent with deep-sequencing results (Figure 2) with the exception of only four miRNAs (rno-miR-296, rno-miR-93, rno-miR-99b and rno-miR-130a), which exhibited minor discrepancy between qPCR and deep-sequencing results at P0. These results further showed the high accuracy of deep-sequencing in detection and quantification of the relative expression levels of most miRNAs. The expression level of one extensively studied miRNA rno-miR-134, which plays important roles in regulation of embryonic stem cell differentiation and synapse plasticity [28-30], was used as a relative standard to judge the abundance of detected miRNAs. The expression levels of rno-miR-134 in our samples were 350.10 and 326.51 TPM at E13 and P14, respectively, and were less than 300 TPM at other stages. We found that there were 50 miRNAs whose expression was >300 TPM at more than one developmental stages, and 162 miRNAs exhibited <300 TPM expression in all developmental stages. This means that although most known miRNAs were detected in cortex, only one-third was abundantly expressed and may play significant roles during cortical development, although other relatively low-abundance miRNAs may also play some roles. The top 20 most abundant miRNAs at each developmental stage are summarized in Table 2.

**Table 1:** Summary of miRNAs from the deep-sequencing results

	**Known miRNAs**	Known miRNAs*	**TPM >300**	**TPM >1000**	**Novel miRNAs**
**E10**	278	56	110	51	40
**E13**	272	53	99	49	30
**E17**	263	54	74	36	27
**P0**	258	54	76	41	24
**P3**	263	54	72	37	24
**P7**	264	54	79	50	11
**P14**	267	54	85	55	12
**P28**	265	54	88	53	18

**Table 2:** The top 20 highly expressed miRNAs at different developmental stages

**E10**	**E13**	**E17**	**P0**	**P3**	**P7**	**P14**	**P28**
rno-let-7c	rno-let-7c	rno-let-7c	rno-let-7c	rno-let-7c	rno-let-7c	rno-miR-128	rno-miR-128
rno-miR-9	rno-let-7a	rno-let-7a	rno-miR-124	rno-miR-128	rno-miR-128	rno-let-7c	rno-let-7c
rno-let-7a	rno-miR-199a-3p	rno-let-7f	rno-let-7a	rno-let-7f	rno-let-7f	rno-let-7f	rno-let-7a
rno-miR-103	rno-miR-103	rno-miR-99a	rno-let-7f	rno-let-7a	rno-let-7a	rno-let-7a	rno-let-7f
rno-let-7f	rno-let-7e	rno-let-7d	rno-miR-128	rno-let-7b	rno-let-7b	rno-let-7d	rno-let-7d
rno-miR-199a-3p	rno-miR-99a	rno-let-7b	rno-let-7b	rno-miR-124	rno-miR-124	rno-let-7b	rno-miR-29a
rno-miR-107	rno-let-7f	rno-miR-9	rno-miR-99a	rno-miR-103	rno-let-7d	rno-miR-124	rno-let-7b
rno-miR-181a	rno-miR-140*	rno-miR-124	rno-miR-9	rno-miR-9	rno-miR-99a	rno-miR-99a	rno-miR-124
rno-miR-101b	rno-miR-107	rno-miR-128	rno-miR-103	rno-miR-107	rno-miR-103	rno-miR-103	rno-miR-103
rno-let-7e	rno-miR-9	rno-miR-103	rno-let-7d	rno-let-7d	rno-let-7i	rno-miR-107	rno-let-7e
rno-miR-124	rno-miR-30d	rno-let-7e	rno-miR-101a	rno-miR-101a	rno-miR-107	rno-let-7e	rno-miR-107
rno-miR-140*	rno-miR-124	rno-miR-125b-5p	rno-miR-107	rno-let-7i	rno-miR-30d	rno-miR-30d	rno-miR-99a
rno-miR-101a	rno-let-7d	rno-miR-107	rno-let-7e	rno-miR-101b	rno-miR-9	rno-let-7i	rno-let-7i
rno-miR-181b	rno-miR-30a	rno-let-7i	rno-miR-125b-5p	rno-miR-181a	rno-miR-101a	rno-miR-9	rno-miR-30d
rno-miR-30a	rno-miR-7a	rno-miR-101a	rno-let-7i	rno-miR-125b-5p	rno-miR-30a	rno-miR-29a	rno-miR-9
rno-miR-181d	rno-miR-320	rno-miR-30d	rno-miR-181a	rno-let-7e	rno-miR-9*	rno-miR-30a	rno-miR-30a
rno-let-7i	rno-miR-191	rno-miR-30a	rno-miR-101b	rno-miR-320	rno-let-7e	rno-miR-101a	rno-miR-101a
rno-miR-320	rno-miR-101a	rno-miR-9*	rno-miR-140*	rno-miR-140*	rno-miR-101b	rno-miR-185	rno-miR-219-2-3p
rno-miR-99b	rno-miR-101b	rno-miR-191	rno-miR-320	rno-miR-99a	rno-miR-185	rno-miR-101b	rno-miR-185

We observed that although there was no obvious difference in the total number of unique miRNAs detected in cortex across different developmental stages, the expression level of different miRNAs in cortex was very dynamic over stages. We carried out the clustering analysis for all detected known miRNAs and 44 novel miRNA candidates (see below) based on their relative expression levels (Figure 2G). Dataset S1 shows a list of these known and novel miRNAs in the order of clustering result. As shown in Figure 2, more miRNAs exhibited higher expression level in earlier developmental stages than later stages. Nearly 40 % of miRNAs had the highest abundance at E10. Moreover, more miRNAs exhibited a higher abundance in early developmental stages (E10 and E13) and late developmental stages (P7-P14) than in middle stages (E17-P3). Overall, the expression patterns of miRNAs fell into four main categories: (1) Enriched in early embryonic stages, especially at E10 and E13 and decreased gradually during development (i.e. the rno-miR-181 family); (2) Enriched late postnatally, especially at P14 and P28, and tended to increase over time (i.e. rno-miR-29a and rno-miR-128); (3, 4) Peaked around neonatal stage (P0), either highest peak or lowest peak.

The expression profile of miRNAs provides a hint of their potential functions during development. For example, at E10, which is a stage of fast proliferation and expansion of cortical progenitor cells, more than 100 miRNAs exhibited higher expression than any other developmental stages. Some of these miRNAs, i.e. rno-miR-34c, rno-miR-449a, rno-miR-301b, rno-miR-532-5p, rno-miR-219-5p, rno-miR-451, and rno-miR-152, were even 10-fold more abundant at E10 than at any other stages, providing a hint that these 7 miRNAs may play important roles in the regulation of progenitor cell proliferation. At about E13, when the first waves of neurons are produced from neural progenitor cells in rat cortex [25], we found that 4 miRNAs were particularly high at this stage, including rno-miR-199a-3p, rno-miR-494, rno-miR-182, and rno-miR-7a, suggesting important roles of these miRNAs in neurogenesis. At neonatal stage (around P0), when the majority of pyramid neurons have already migrated to their destinations and are extending axons and dendrites [31], we found high expression of several miRNAs at this stage, i.e. rno-miR-137 and rno-miR-19b. Consistently, a previous study showed that miR-137 regulates neuronal maturation by targeting the ubiquitin ligase Mib-1 [32]. Dataset S1 provides a complete list of the name and relative abundance (TPM) of all detected known miRNAs.

We note that during the preparation of this manuscript, one group reported the identification of two novel miRNAs from the brain tissue named as rno-miR-344b-5p and rno-miR-3559-5p [33]. Our work further verified their finding of these two novel miRNAs in brain tissues (Figure S3). The expression of rno-miR-344b-5p gradually elevated during development, suggesting that its function may involve late developmental processes like the synapse development and plasticity [34]. Expression of rno-miR-3559-5p dropped over development, with a peak at E13, suggesting a potential role in embryonic neurogenesis.

### Identification of potential novel miRNAs in cortex

**Figure 3:**
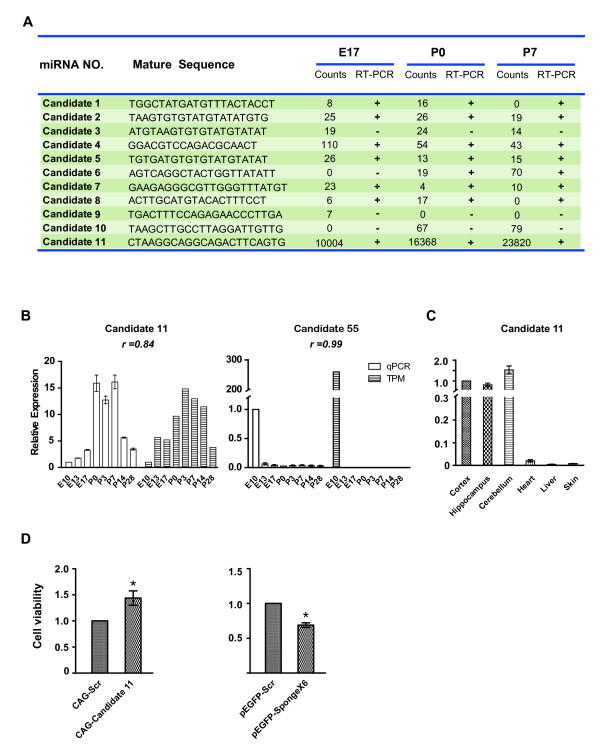
**Validation of the expression of novel miRNAs.A.** Summary of the RT-PCR analysis of the 11 selected novel miRNAs. Novel candidates with extremely lower abundance can be detected by deep-sequencing but not by PCR amplification. Counts mean the numbers of each unique read coming from deep sequencing. **B.** Validation of the expression profile of 2 highly expressed novel miRNA candidates (candidate 11, candidate 55) by qPCR. All experiments were repeated for 3 times. Error bars represent standard deviation. The relative expression was normalized to E10 value for both qPCR and TPM. There is a high correlation between the deep-sequencing and qPCR results. **C.** Expression of the candidate in different tissues. The relative expression of miRNAs in different tissues was normalized to that of cortex. Note that all of them are enriched in the central nerve system. Experiments were repeated for 4 times. Error bars represent standard error deviation (n = 4; *P < 0.05). **D.** Regulation of C6 cell proliferation by Candidate 11. Overexpression of Candidate 11 promoted cell proliferation, whereas suppressing the endogenous Candidate 11 by overexpression of a sponge RNA (6X) reduced cell proliferation. Experiments were repeated for 3 times. Error bars represent standard error of the mean (n = 3; *P < 0.05).

One advantage of deep-sequencing in miRNA detection is its ability to discover potential novel miRNAs. In the current study, the miReap algorithm [35] (Http://sourceforge.net/projects/mireap) was employed to call all candidate miRNA precursors with hairpin-like structures (see methods). Altogether, 101 potential novel miRNAs were identified in this study when annotated to the miRBase release 18.0. Dataset S2 provides a complete list of the name and relative abundance for all novel miRNA candidates based on annotation to release 18.0 of miRBase. The predicted structure of 11 newly identified miRNAs are shown in Figure  S4 as examples. The existence of these 11 novel candidates was further verified by RT-PCR, together with several recently identified miRNAs (rno-miR-2964, rno-miR-344b-5P, and rno-miR-3559-5p). We found that those with extremely low reads failed to be consistently detected using PCR (Figure 3A). Overall, eight of the 11 novel candidates were verified by PCR (Figure S5). The expression pattern of 2 highly expressed novel candidates were also verified using qPCR with consistent results as that of deep-sequencing (Figure 3B-D). The number of potential novel miRNAs detected by deep-sequencing was very diverse over development (Table. 1), and the expression level of most novel candidates was very low. Out of the total 101 novel candidates, only 2 candidates were expressed at a relatively high abundance and were thus more likely to play important biological functions in brain. Among these 2 candidates, Candidate 55 was enriched at E10 (259.55 TPM), and was not detected in any other developmental stages. The expression level of the Candidate 11 reached a peak at P3, a stage characterized with the peak of gliogenesis in rat cerebral cortex [36]. Next, we compared the expression level of Candidate 11 in different tissues including cortex, hippocampus, cerebellum, skin, heart, and skin. We found that this novel candidate was enriched in central nerve system (Figure 3C).

To test whether the biogenesis of novel candidates depends on Dicer, we compared the expression level of mouse homologues of candidate novel miRNAs in cortical tissue of wild type mice and mutant littermates of brain-specific knockout of Dicer (Nestin-Cre, Dicer-floxed). As positive control, the expression of three known miRNAs, miR-134, miR-124, and the newly identified miR-344b-5p, was significantly reduced in Dicer knockout brain. The expression levels of mouse Candidate 11 also significantly decreased in homozygous knockout brains (Figure S6), further supports the notion that it indeed belongs to the category of miRNA.

Dataset S2 provides a complete list of the name and relative abundance (TPM) for all detected novel miRNAs, some of which were selected for clustering analysis together with known miRNAs (Figure 2G; Dataset S1). To get functional hints for these novel candidates, we predicted the potential target genes of these miRNAs by using TargetScan [37], and carried out gene ontology (GO) enrichment analysis with GOrilla of predicted target genes (Dataset S3). We found that the potential function of Candidate 11 may be involved in regulating energy production and G-protein-coupled receptor signaling pathway. Considering that Candidate 11 has highest expression at P3, which is a peak stage for gliogenesis in cortex [36], we further examined whether it affects the proliferation of glial cells using cultured rat C6 glial cell line. Interestingly, overexpression of Candidate 11 in C6 cells increased the cell proliferation, whereas suppressing the endogenous Candidate 11 by overexpressing a specific sponge RNA [38] reduced the cell proliferation (Figure 3D). This result supports the notion that this novel miRNA may regulate the gliogenesis during cortical development.

### Potential stage-specific RNA modification during cortical development

Recent studies showed that miRNAs may undergo cleavage at the 3’ end by specific exoribonuclease, resulting in the existence of multiple isoforms of variant lengths (isomiRs) [39]. We note that in all cortical RNA samples, variability in the length of miRNAs was detected as addition and/or trimming of nucleotides at both 3’ end and 5’ end of mature miRNAs. Majority of known miRNAs underwent trimming at both 3’ and 5’ ends. However, trimming for several miRNAs including rno-miR-1*, rno-miR-196a*, rno-miR-207, rno-miR-347, and rno-miR-742 was not detected, possibly due to the low abundance of trimmed isoforms rather than a selective protection of modifications. Consistent with previous findings in Drosophila [40] and in Human[41], we found that 3’ end trimming is the most frequent type of isomiR in all cortical samples (Figure S7). This also suggests that there is no stage-specific regulation of the trimming of miRNAs. Dataset S4 provides a complete list of the name and relative abundance (TPM) of all detected isomiRs of known miRNAs.

**Figure 4:**
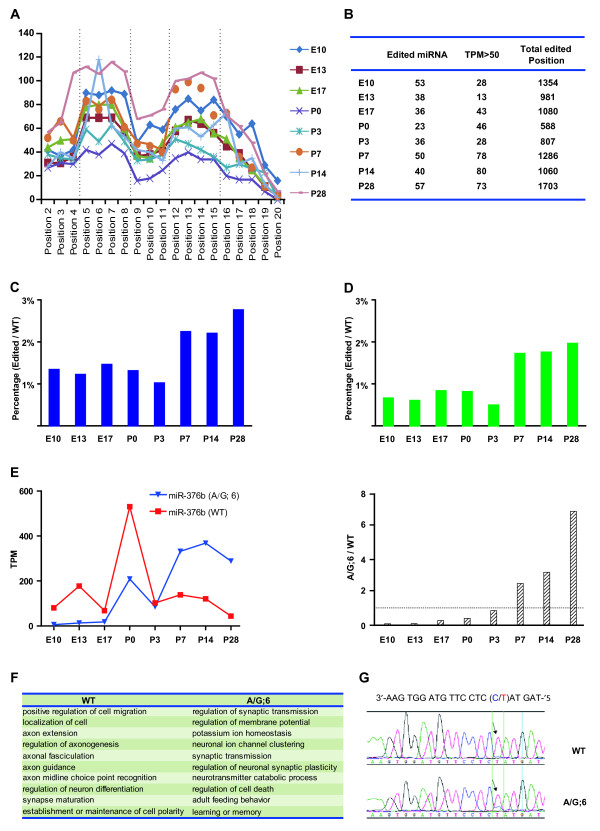
**Characterization of miRNA editing during cortical development.A.** Cumulative counts (TPM) of editing at each position of all mature miRNA sequences at different developmental stages. The editing events were enriched at “seed” sequence (position 2 to 8) and flanking region (position 12 to 15) of miRNAs. **B.** Summary of miRNA editing at different stages detected by deep-sequencing. There are more edited miRNAs and modified positions at P28 than other stages. The number of highly edited miRNAs (TPM > 50) gradually elevated during development. **C-D:** The percentage of total edited miRNA reads among total miRNA reads was much higher after P7 than earlier stages (**C**). Similar tendency was also observed for miRNAs of high editing events (>50 TPM) (**D**). **E.** Developmental changes of the expression of wild type (WT) and “A” to “I” edited miRNA-376b at position 6 (A/G: 6). The level of edited form exceeded that of WT from P7. **F.** Summary of biological processes that may be regulated by WT and edited (A/G:6) miRNA-376b. All target genes of the wild type and edited miRNAs were predicted by TargetScan and statistically over-represented GO annotations were included (*P* < 0.01). Note that GO terms of WT are those involved in early development of cortex. Those GO terms unique to edited miRNA targets are associated with establishment and maintenance of neural circuitry. G. Validation of “A” to “I” editing of miRNA-376b at position 6. RNAs from P7 rat cortex were reverse transcribed and then amplified by PCR. The PCR product was sequenced. A heterozygous change A-G was detected at position 6 (arrow).

RNA editing has emerged as one important posttranscriptional mechanism that introduces nucleotide changes in RNA sequence, such as cytidine (C) to uridine (U) and adenosine (A) to inosine (I) via deamination [42], and may play important regulatory roles in the nervous system [43]. Although the majority of editing events happens to pri-miRNA and appear to affect the miRNA processing step, some nucleotide alterations happen in the “seed” sequence of mature miRNAs [44]. These edited mature miRNAs with altered “seed” sequence are likely to suppress a set of genes different from those targeted by unedited miRNAs. We systemically examined the nucleotide changes of mature miRNAs by alignment of unannotated tags with mature sequence of miRNAs allowing one nucleotide mismatch [45,46]. We discovered 160 miRNAs with single nucleotide modification located across the mature sequence with obviously higher frequency of modification detected at the “seed” and flanking regions (Figure 4A). The existence of such a peak in nucleotide changes at “seed” and flanking sequences suggests that most observed nucleotide changes were not caused by random mutations during sequencing, but by active miRNA editing in the cell. Besides the well-known “A” to “I” modification, many other RNA editing events were also discovered such as “A” to “C” and “G” to “T” (Dataset S5), consistent with a widespread RNA editing discovered in previous human transcriptome studies [47-49]. Although the expression level of the majority of edited miRNAs was very low, some particularly high frequent editing events happened at certain developmental stages. Taking rno-miR-128 as an example, highest frequency of “A” to “C” editing at position 3 and “G” to “T” editing at position 6 was observed at P14, whereas “G” to “T” editing at position 8 was highest at P3 (Figure S8).

We found that the number of miRNAs with a relatively high editing events (>50 TPM) was much higher after P7 than at earlier developmental stages (Figure 4B). Moreover, the percentage of total edited miRNA reads among total miRNA reads was also much higher after P7 than earlier stages (Figure 4C). Similar tendency was observed for miRNAs of high editing events (>50 TPM) (Figure 4D). These results suggest the necessity of miRNA editing for complex regulation of gene expression at late postnatal stages, potentially contributing to the complicated synaptic wiring. As a distinguished representative of miRNA editing, rno-miRNA-376 family have been extensively studied [5]. The previously reported “A” to “I” editing at position 6 (A/G: 6) of rno-miRNA-376b was also detected in the present study by both deep-sequencing and PCR-based sequencing (Figure 4G). Deep-sequencing results showed that the level of this “A” to “I” editing at position 6 of rno-miRNA-376b increased during cortical development. Surprisingly, the expression level of edited sequence exceeded that of the wild type form from P7 and reaches the peak at P28 (Figure 4E), indicating that the edited sequence may play important roles in late postnatal development of cortex. To further understand the biological significance of this editing event of rno-miR-376b, target prediction and GO analysis was introduced (Dataset S6). We found that the potential function of wild type rno-miR-376b may be mainly related to early developmental events including neuronal differentiation, cell migration, axon extension, and establishment or maintenance of neuronal polarity. However, the potential function of the edited isoform shifted to the regulation of late developmental events including synaptic plasticity, learning and memory, and adult feeding behavior (Figure 4F, Dataset S6). Interestingly, results of this GO analysis are fully consistent with the high expression of the wild type rno-miR-376b and the edited isoform at early developmental stages and late postnatal stages, respectively. Dataset S5 provides a complete list of the name and relative abundance (TPM) for all detected editing of miRNAs, with TPM >100 highlighted.

## Discussion

Accumulating evidences showed that different groups of small non-coding RNAs play fundamental roles in gene regulatory networks. As the most abundant group of small RNAs in many tissues, miRNAs play important regulatory roles in physiological and developmental processes. In the nervous system, miRNAs can also function as important mediator of various pathological processes [50]. Recently, exogenous expression of miR-9/9* and miR-124 in human fibroblasts was shown to convert these cells into neurons [51,52], suggesting the wide application potential of miRNAs. Here, we took advantage of high-throughput sequencing technology to quantitatively analyze the expression of miRNAs in rat cortical tissues of many developmental stages. We found that miRNAs showed a wide diversity of expression pattern during cortical development. Some miRNAs seem to be preferentially enriched in early embryonic cortex, whereas others exhibited a higher abundance in postnatal tissue, indicating distinct roles played by these different groups of miRNAs in controlling cortical development. The expression patterns of some miRNAs observed in our study are consistent with what were observed in previous studies by using the blot-array and Northern blot assays, i.e. miR-125b, miR-9, and miR-181a [6], as well as miR-29a, miR-138 and miR-92 [53]. We note that the developmental expression pattern of miRNAs provides a hint of their potential functions. The dataset described here will thus provide an enriched resource for searching miRNAs that may play key regulatory roles at different stages of cortical development. In support of this notion, we observed that the novel miRNA Candidate 11 promoted the proliferation of cultured C6 glial cells, consistent with the high expression of this miRNA around the peak stage for gliogenesis in cortex (P3). It would also be very interesting to explore whether the expression of this novel miRNA correlates with and contributes to the happening of glioma in human patients.

One recent study reported strain-specific miRNAs in rats. The authors provided an in-depth analysis of small RNA profiles of six different tissues (spleen, liver, brain, testis, heart, kidney) of two different rat strains (BN-Lx and SHR) [33]. We found that the majority of miRNAs they discovered can be confirmed in our study. Several miRNAs including rno-miR-582, rno-miR-666-3p, and rno-miR-2985-3p were not detected in our study. In contrast, several E10-enriched miRNAs identified in our study, including rno-miR-181a, rno-miR-449a, and rno-miR-503, were not detected in their results. These differences in miRNA detection may due to the failure of detection of some low abundance ones in different studies. The existence of strain-specific expression of several miRNAs may also be responsible for the differential detection in different studies. Moreover, we detected the expression of low abundance miRNAs that have not been detected before using other techniques. One example is miR-128, which was reported to be specifically expressed in postnatal cortex [6]. However, our results showed that miR-128 was also expressed in embryonic cortex with much lower abundance, indicating that high throughput sequencing is much more sensitive than conventional methods.

Besides the identification of dozens of novel miRNAs at each developmental stage, we observed in developing cortex extensive RNA editing in the miRNA “seed” and flanking sequences. Since most nucleotide changes at specific position of miRNAs was detected up to hundreds or even thousands of times, and the relative abundance of certain modified miRNAs at different developmental stages was not proportional to that of the wild type miRNAs, it is unlikely that the nucleotide changes we observed were caused by random errors during sequencing. The high tendency of nucleotide changes at “seed” and flanking sequence also supports the existence of a highly-regulated “editing” process (Figure 4B). We found that the predicted target genes of the wild type rno-miR-376 and the “A” to “I” edited isoform are of totally different functional groups (Figure 4F). Interestingly, the relative abundance of “A” to “I” editing of rno-miR-376 gradually increased during development and surpassed that of wild type isoform at P7, indicating that RNA editing may be a new strategy for the regulation of gene expression during brain development.

Previous study showed that adenosine deaminases (ADARs) catalyze the “A” to “I” editing of RNAs [54]. Editing of glutamate receptor by ADARs is involved in neural development and diseases [55,56]. Cytidine deamination by members of the apolipoprotein B mRNA editing complex polypeptide 1-like (APOBEC) family of enzymes has also been shown to be an important mechanism for the silencing of retrovirus and transposable elements [57]. Interestingly, our preliminary study showed that both ADAR and APOBEC family members could be detected in developing cortical tissue (data not shown). For the miRNA editing in developing cortex, a number of questions remain to be clarified in the future: (1) Are ADAR and/or APOBEC family proteins responsible for the different types of editing of cortical miRNAs? (2) Are there other enzymes contributing to the miRNA editing in cortex? (3) How the nucleotide-specificity of the editing is achieved? (4) How is the miRNA editing regulated by intracellular signal cascades during development? Extensive experimental studies are required in the future to address these questions.

Previous studies showed that rasiRNAs and piRNAs are of the same origin, yet with slight differences in the way of identification and nomenclature. [58]. The rasiRNAs were first defined as small RNAs derived from repeat elements, mainly transposons, in the genome [59,60]. However, piRNAs were first identified as small RNAs associated with PIWI proteins in germline tissues [61]. Later studies showed that both rasiRNAs and piRNAs are derived from repeat elements [62] and serve to suppress the activity of transposable elements by guiding the epigenetic silencing of the transcription of transposable elements and by guiding the direct cleavage of transcripts of these transposons [63]. Recently piRNAs were detected in adult cerebral cortex of rat and showed altered expression after transient focal ischemia [64]. Nearest, piRNAs were reported as functional regulator of enhancing long-term synaptic facilitation through silencing CREB2 [65]. Here we detected a low-abundance expression of a group of piRNA-like small RNAs in developing cortex of rat based on the sequence mapping to reference libraries (piRNA Bank, http://pirnabank.ibab.ac.in/index.shtml) [66]. Moreover, we observed in cortical tissues the expression of PIWI-like proteins (data not shown), which play important roles in the biogenesis and function of piRNAs or rasiRNAs [58], further supporting the existence of piRNAs or rasiRNAs in brain. Interestingly, recent studies showed that retrotransposable events actively happen during neurogenesis and may contribute to the diversity of neuronal phenotypes [67,68]. Since we observed much higher rasiRNA level at early developmental stages than in the adult, an intriguing possibility is that rasiRNAs in developing cortex may also contribute to the maintenance of the genome stability in neural progenitor cells by suppressing the mobile elements, a potential mechanism that deserves to be further addressed by experimental studies in the future.

## Conclusion

High-throughput sequencing provides a good opportunity to systematically analyze the transcriptome of small RNAs of cortical tissues. In this study the use of this technique led to the quantitative clarification of the expression of a large number of previously un-detected small RNAs in cortical tissues, including miRNAs, rasiRNAs and/or piRNA-like RNAs, and small RNAs derived from rRNA, tRNA, snoRNA, snRNA, and scRNA. We demonstrated dynamic and stage-specific expression of a large group of known miRNAs, with surprisingly profound nucleotide editing at “seed” and flanking sequences of miRNAs during cortical development. In addition, we identified a group of novel miRNA candidates in rat cortex with functional hints. The dataset described here will be a valuable resource for clarifying the gene regulatory network during brain development and disease.

## Methods

### Animals

All rats and mice used in the present study were provided by Shanghai SLAC Laboratory Animal Co. Ltd. Experimental procedures involving animals were carried out under the guideline and permission of the Animal Care and Use Committee of the Institute of Neuroscience at the Shanghai Institute for Biological Sciences, Chinese Academy of Sciences (NA-100410-4).

### RNA extraction, construction of small RNA libraries, and deep-sequencing

Rat (Sprague–Dawley) cortical tissues of various developmental stages were quickly harvested on ice. For E10 and E13 brains, the whole cortex tissues were collected. For E17-P28 brains, the dorsal lateral regions of the cortex, mainly the somatosensory cortex, were collected. Subcortical tissues and meninges were carefully removed under dissecting microscope. For collection of cortical tissues of wild type and Dicer knockout mice, Dicer-floxed mice were crossed with the Nestin-Cre line to knockout Dicer in brain. E16 cortical tissues of wild type and homozygous mutant embryos were dissected under microscope. Total RNA was then extracted with TRIzol reagent (Invitrogen, Carlsbad California) following the manufacturer’s instruction. The RNA integrity number (RIN), an algorithm for judging the integrity of RNA samples, were evaluated using Agilent 2100 Bioanalyzer (Agilent Technologies, Paloalto, USA) following the manufacturing instruction. Size-fractionation was performed on 15% polyacrylamide gel electrophoresis (PAGE) to collect the 10–35 nt fraction.

Small RNA library construction and deep sequencing were carried out by BGI (Beijing Genome Institute at Shenzhen, China). Briefly, adapters were ligated to the 5’ and 3’ termini of these small RNAs, which then were used as templates for cDNA synthesis. After producing libraries via PCR amplification, purified PCR products were then sequenced using the Solexa 1 G Genome Analyzer to get 35 nt reads. After filtering out low quality reads, trimming the adapter sequence, cleaning up contaminants formed by ligation, clean reads of 18–30 nt were grouped and used for further analysis.

### Computational analyses

Clean reads of unique small RNA tags were counted as their expression abundances. Those identical RNA tags were mapped to rat genome (Baylor 3.4/rn4) by SOAP software (Short Oligonucleotide Alignment Program, http://soap.genomics.org.cn) to analyze the expression of corresponding small RNA genes and their distribution on the genome. Small RNA tags were aligned to the miRNA precursor and mature sequences from miRbase 18.0 (www.miRBase.org) to obtain the known miRNA counts. Unannotated tags were aligned to the sequences of other class of non-coding RNAs (rRNA, tRNA, scRNA, snRNA, snoRNA and piRNA) from Rfam [69,70] (http://www.sanger.ac.uk/software/Rfam) and the GenBank (http://www.ncbi.nlm.nih.gov/). The read count of each unique tag was normalized to transcripts per million (TPM), according to the total read count.

To identify potential novel miRNAs, the software Mireap (http://sourceforge.net/projects/mireap) was used to explore the secondary structure, the Dicer cleavage site, and the minimum free energy of the unannotated small RNA tags which could be mapped to genome. In brief, the sequence length should be between 18–26 nt, maximal free energy allowed for a miRNA precursor was ~18 kcal/mol, maximal space between miRNA and miRNA* was 35 nt, and flanking sequence length of miRNA precursor should be 10 nt. After filtering in above analysis pipeline, unannotated small RNA tags were aligned with mature miRNAs from miRBase18.0 to detect miRNA editing allowing one mismatch on certain position of miRNAs. To eliminate sequence changes generated by single-nucleotide polymorphism (SNP) at the genomic DNA, the results were filtered with SNP database (Build 130) (http://www.ncbi.nlm.nih.gov/projects/SNP/index.html). IsomiR analysis was conducted by aligning the reads to precursor sequence and mature sequence of miRNAs. IsomiRs were divided into 8 groups as follows: 1, Addition of nucleotides at both 3’ and 5’ ends; 2, Addition of nucleotides at 5’ end; 3, Addition of nucleotides at 3’ end; 4, Addition at 5’ end and trimming of nucleotides at 3’ end; 5, Trimming at 5’ end; 6, Trimming at both 3’ and 5’ ends; 7, Trimming at 3’ end; 8, Trimming at 5’ end and addition at 3’ end. Pearson’s correlation algorithms were used to assess the correlation between read counts per miRNA of the two P0 samples.

### Clustering analysis and heat map presentation

Heat map about relative abundances of different classes of small RNAs was done as follows. All abundance values are normalized by the E10 value and colored in terms of significance, with green indicating that abundance of a term is significantly lower than average, and red indicating higher than average [71]. Over-representation for each term in a group is calculated as follows:

(1) $$\text{Computation formula}:\text{Z}=\left(\text{X}-\text{Avg}\right)/\text{SD}\text{.}$$

Where *X* is the abundance of a term in the group being considered, *Avg* is the average abundance of a term in all developmental stages, and *Z* presents the relative abundance of a term at a given developmental stage. The Complete Linkage Clustering of known and novel miRNAs was obtained based on Hierarchical Clustering Algorithms by using the R package [72].

### Target prediction and gene ontology analysis

The potential target genes were predicted by TargetScan [37] and then assayed by Gorilla [73] with gene ontology (GO) enrichment analysis (http://cbl-gorilla.cs.technion.ac.il/).

### Quantitative RT-PCR

Reverse transcription reactions were performed in a final volume of 20 μl containing 2 μg purified total RNA, 1 × RT buffer (Promega, USA), 10 mM dNTPs, 5 U M-MuLV reverse transcriptase (Promega, USA), 20 U RNase inhibitor (Fermantas, USA) and 0.4 μM stem-loop RT-primers. The reactions were incubated in Thermo Cycler (BioRad, USA) at 37°C for 60 min, 90°C for 5 min and then held in 4°C.

Realtime PCR was performed on 7500 Fast Real-time PCR system (Applied Biosystems,USA)(27). In brief, reactions were performed in a final volume of 20 μl containing 10 μl SYBR ® Green Master mix, 1 μl RT products (diluted for 10 times), 1 μM unique primer of certain miRNA, and 1 μM out-primer match to the stem-loop sequence. PCR reaction was carried out with a first denaturation step at 95°C for 20 sec, followed by 45 cycles comprising denaturation at 95°C for 12 sec, annealing and extension at 56°C for 30 sec. Melting curve was run in program following 95°C, 15 sec; 60°C, 20 sec; 95°C, 15 sec; 60°C, 15 sec. To normalize the differences of the amount for different samples, U6 was used as internal control as well as experimental positive control. Negative controls (without template) were also established and all experiments were run in triplicate. The 2_T_^-ΔΔC^ method was applied for relative expression quantification analysis [74] and E10 value was used as reference. All PCR products were cloned into pGEM-T vector (Promega, USA) and then sequenced. Primers used are shown in Dataset S7.

### PCR analysis

For PCR verification of novel miRNAs, reverse transcription was performed with Revert Aid First Strand cDNA Synthesis kit (MBI Fermentas) using specific stem-loop primer (Dataset S7). PCR was carried out with a first denaturation step at 95°C for 3 min, followed by 35 cycles comprising denaturation at 95°C for 20 sec, annealing at 60°C for 25 sec, and extension at 72°C for 45 sec. PCR products were separated by agarose electrophoresis.

For PCR analysis of Piwi expression, synthesis of first strand cDNA was carried out with a Revert Aid First Strand cDNA Synthesis kit (MBI Fermentas). PCR was carried out using cDNA with the following protocols: Initiate denaturation at 94°C for 5 min; denaturation at 94°C for 45 sec, annealing at 62°C for 30 sec, extension at 72°C for 45 sec, and a 10 min 72°C final extension. Cycle numbers for actin, Piwil1, 2, and 4 were 25, 35, 42, and 42. Predicted sizes for PCR products for Piwil1, 2, and 4 are 178 bp, 152 bp, and 179 bp, respectively. Primers used are shown in Dataset S7.

For PCR verification of editing of miR-376b, genomic DNA and cDNA from P7 rat cortex were used as template. PCR was performed as following protocols: Initiate denaturation at 94°C for 5 min; denaturation at 94°C for 45 sec, annealing at 60°C for 30 sec, extension at 72°C for 45 sec, PCR program was run for 35 cycles, with a 10 min 72°C final extension. PCR products were treated with SAP (1U) and Exonuclease I (20U) (Takara, Japan) and then subjected to direct DNA sequencing in both directions using forward and reverse primer with an ABI PRISM 3100-Genetic Analyzer (Applied Biosystems). Sequenced PCR products were aligned to precursor sequence of miR-376b using the DNAstar program. Primers used are shown in Dataset S7.

### Plasmid construction and cell proliferation assay

The genome locus of novel Candidate 11 was amplified using PCR and subcloned into ClaI/XhoI site of the pCAG-IRES-EGFP vector. For construction of sponge inhibitor, the synthesized nucleotides with 6 tandem repeat sequence complementary (6X) to mature sequence of Candidate 11 was annealed and cloned into pEGFP-C1. Cell proliferation assay was performed as described previously [75]. Briefly, C6 glial cells were prepared as cell suspension of 50,000 cells/ml in DMEM with 10% fetal bovine serum (Gibco) and transfected with different constructs (3 μg) using the Amaxa Nucleofector kit following the protocol provided by the manufacturer. Each well of a 96-well plate was added with 100 μl of the cell suspension. Culture plate was incubated for 44 hr at 37°C and then added 10 μl CCK-8 solution (Dojindo, japan) into each well, and incubated the plate for another 4 hr at 37°C followed reading the OD at 450 nm to determine the cell viability in each well.

## Abbreviations

E, embryonic day; P, postnatal day; miRNA, microRNA; piRNA, Piwi-interacting RNA; qPCR, quantitative polymerase chain reaction; RIN, RNA integrity number; hr, hour; min, minute; sec, second.

## Competing interests

The authors have no conflict of interest.

## Authors’ contributions

M-J Yao designed the research, conducted most of the data analysis, and wrote the manuscript, G Chen, P-P Zhao and J Jiang contributed to part of data analysis, M-H Lu carried out the qPCR experiments, M-F Liu contributed to experiment design, and X-B Yuan designed the research, supervised the project, and wrote the manuscript. All authors read and approved the manuscript.

## Supplementary Material

Additional file 1 Figure S1. **RNA integrity number (RIN) of all samples.** Electropherograms and calculated RINs of each RNA sample are shown. 18 S and 28 S ribosomal fractions are indicated in pink and dark green colors, respectively. Note that RIN values are between 8.4 and 10, indicating high quality of RNA samples. Click here for file

Additional file 2 Figure S2. **Length distribution of small RNA reads.** The length distribution of small RNA reads for each sample is shown in the histogram. Only reads of 18–30 nt that mapped to rat genomic sequence were included. Note the 22 nt peak in all samples. Click here for file

Additional file 3 Figure S3. **Validation of the expression of miR-344b-5p and miR-3559-5p.** The expression of miR-344b-2 gradually elevated during development. Expression of miR-3559-3p dropped over development, with a peak at E13. These two miRNAs highly enriched in central never system. Click here for file

Additional file 4 Figure S4. **Predicted structures of newly identified miRNAs.** Computationally predicted secondary structures of the primary miRNA transcripts of 11 selected novel miRNA candidates are shown. Mature miRNA sequences are shown in the red frame. Click here for file

Additional file 5 Figure S5. **PCR detection of novel miRNA candidates.** Three known miRNAs, miR-2964, miR-344b-5p, and miR-3559-5p were also included as control. Click here for file

Additional file 6 Figure S6. **Detection of mouse homologue of Candidate 11 in cortical tissue of mutant mouse with brain-specific knockout of Dicer.** A. Genotyping of mutant mice. Nestin-Cre allele generated one band. Heterozygous Dicer-floxed allele generated two bands and homozygous allele generated one upper band. B. Expression level of novel Candidate 11 in P0 cortical tissue of Dicer knockout (Nestin-cre/Dicer-floxed+/+) mice revealed by qPCR. Expression level of Candidate 11 significantly decreased in knockout mice. C. Expression level of three known miRNAs, miR-344b-3p, miR-124, and miR-134, in P0 cortical tissue of wide type and Dicer knockout mice revealed by qPCR. Expression level of the three known miRNAs was remarkably decreased in knockout mice. Click here for file

Additional file 7 Figure S7. **Summary of potential isomiRs in cortical tissues.** A. Summary of the fraction of each major group of isomiR in cortical tissue. Variability in the length of miRNAs was detected as addition and/or trimming of nucleotides at either 3’ end or 5’ end of mature miRNAs. Note that the 3’ end trimming is the most frequent type of modification in all samples. B. A summary of the sequence, length, and count of each isomiR of miR-128 in P14 cortical tissues. Click here for file

Additional file 8 Figure S8. **Detail editing profile of miR-128 during cortical development.** A summary of the position, sequence, abundance (TPM) of each detected editing of miR-128 is shown. The high-abundance edited positions are highlighted with red color. Click here for file

Additional file 9 Table S1. Summary of reads from the deep-sequencing results. Click here for file

Additional file 10 Dataset S1. List of the name and relative abundance (TPM) for all known miRNAs and novel miRNAs. Click here for file

Additional file 11 Dataset S2. **List of novel miRNA candidates.** The name and relative abundance (TPM) for all novel miRNA candidates are shown. List of 44 selected novel miRNA candidates and precursor sequences of 11 selected novel miRNA candidates are also included. Click here for file

Additional file 12 Dataset S3. Predicted targets and GO annotation of three novel candidates. Click here for file

Additional file 13 Dataset S4. **List of the name and relative abundance for all detected isomiRs.** The name and relative abundance (TPM) of isomiRs for all known miRNAs in different samples are shown. Click here for file

Additional file 14 Dataset S5. **Summary of all detected editing of miRNAs.** List of the name, position, and relative abundance (TPM) for detected editing of miRNAs at each developmental stage is shown. High abundant editing is highlighted in yellow. Click here for file

Additional file 15 Dataset S6. Predicted targets and GO annotation of wild type and edited rno-miR-376b (A/G: 6). Click here for file

Additional file 16 Dataset S7. All primers used in present study. Click here for file
