# Supplementary figures and images for: Transcriptome analysis of microRNAs in developing cerebral cortex of rat
Source: BMC Genomics. 2012 Jun 12;13:232. doi: 10.1186/1471-2164-13-232 (PMC3441217; doi:10.1186/1471-2164-13-232)

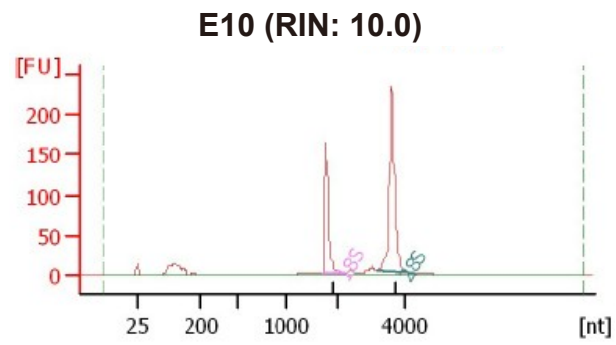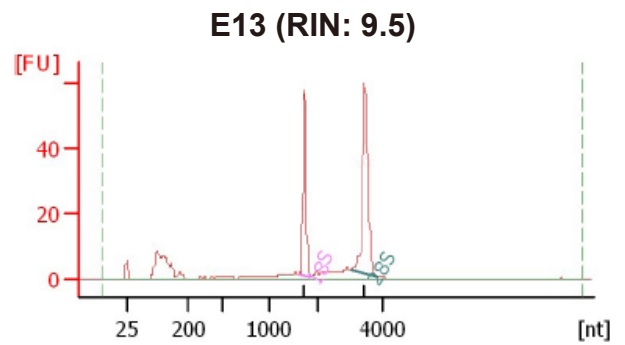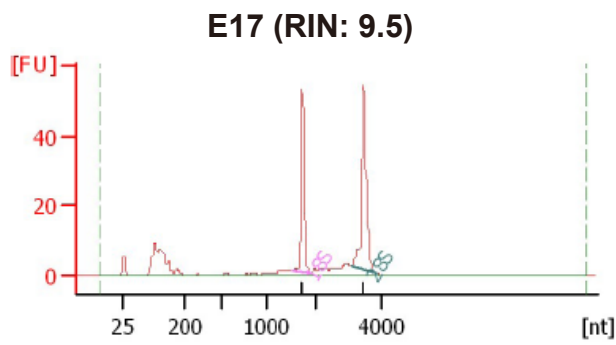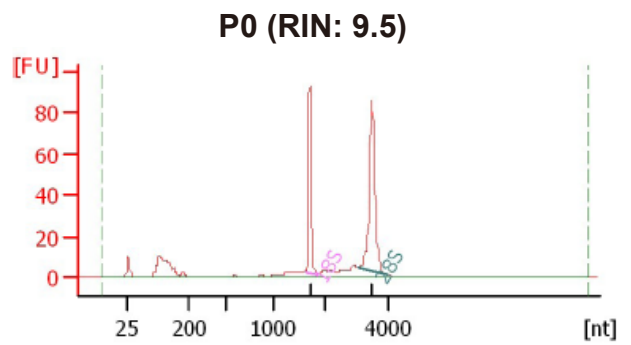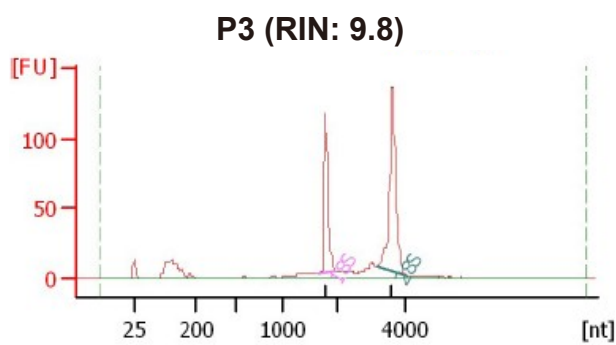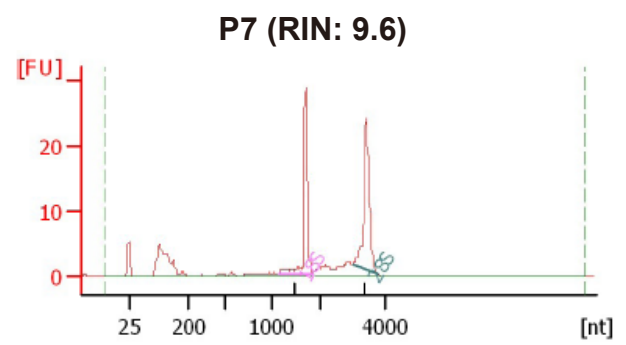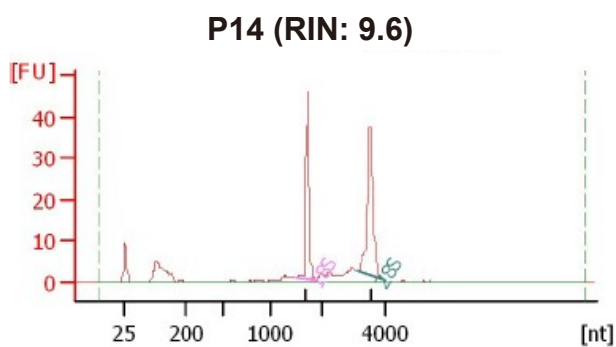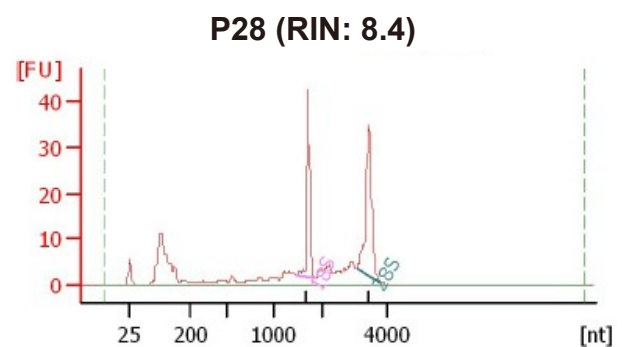

Supplement: Additional file 1 Figure S1. — RNA integrity number (RIN) of all samples. Electropherograms and calculated RINs of each RNA sample are shown. 18 S and 28 S ribosomal fractions are indicated in pink and dark green colors, respectively. Note that RIN values are between 8.4 and 10, indicating high quality of RNA samples. [file 1471-2164-13-232-S1.pdf]

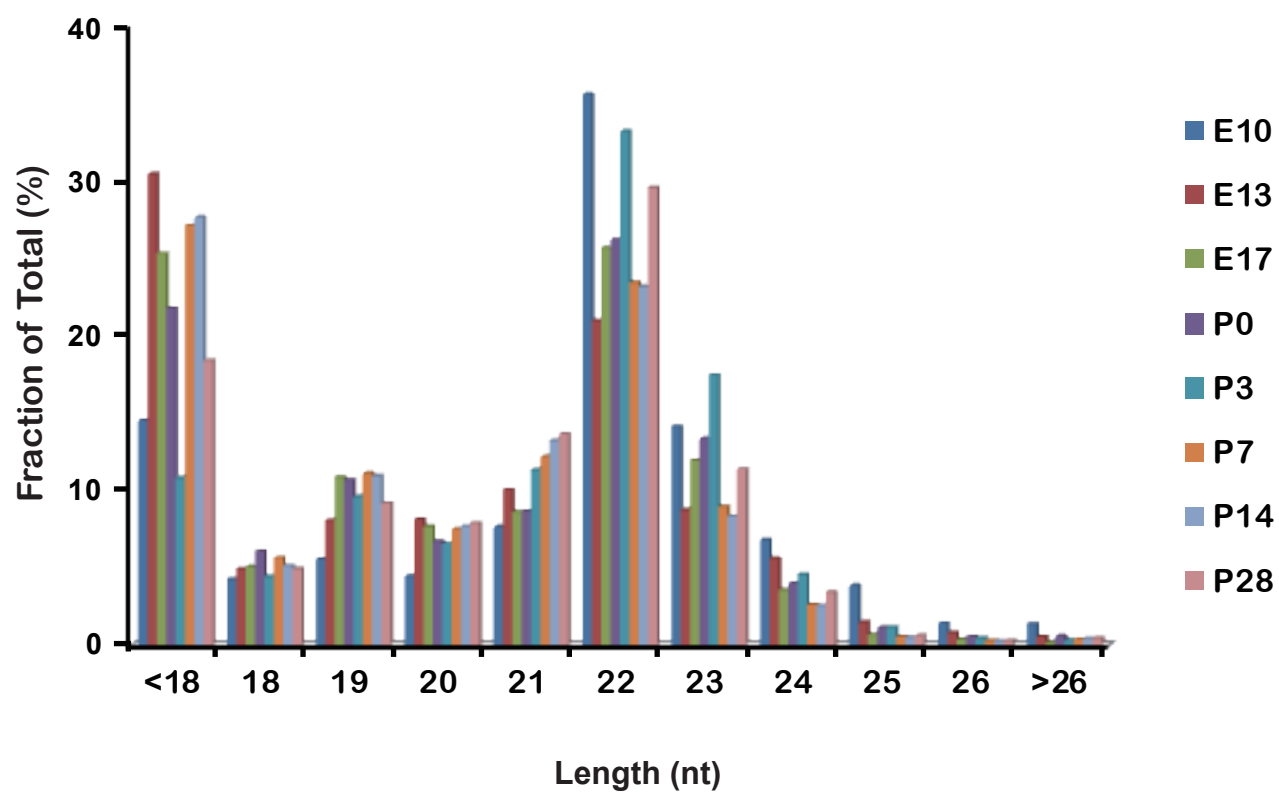

Yao et.al;Fig. S2

Supplement: Additional file 2 Figure S2. — Length distribution of small RNA reads. The length distribution of small RNA reads for each sample is shown in the histogram. Only reads of 18–30 nt that mapped to rat genomic sequence were included. Note the 22 nt peak in all samples. [file 1471-2164-13-232-S2.pdf]

**A**

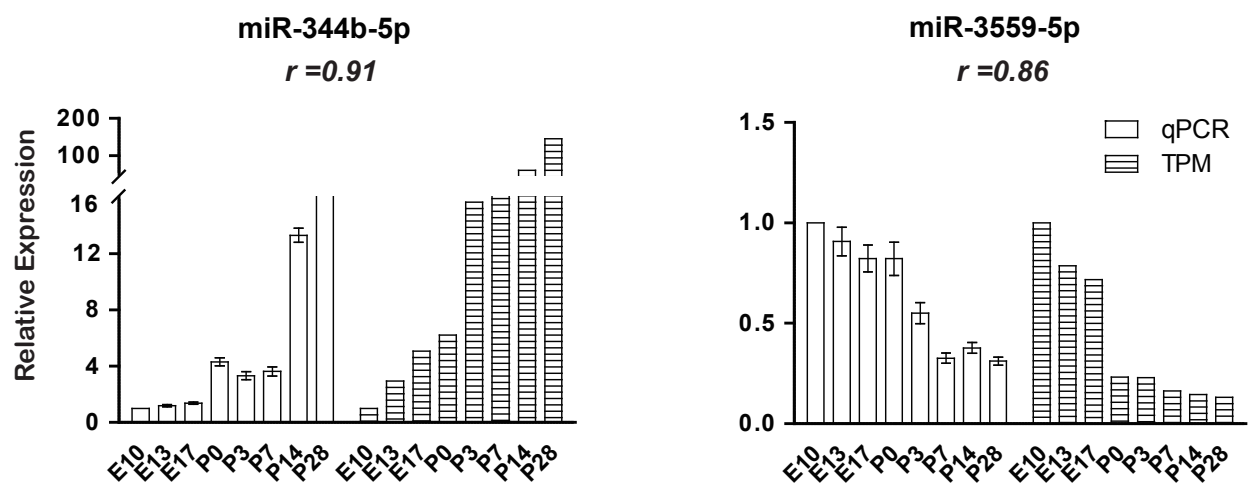

**B**

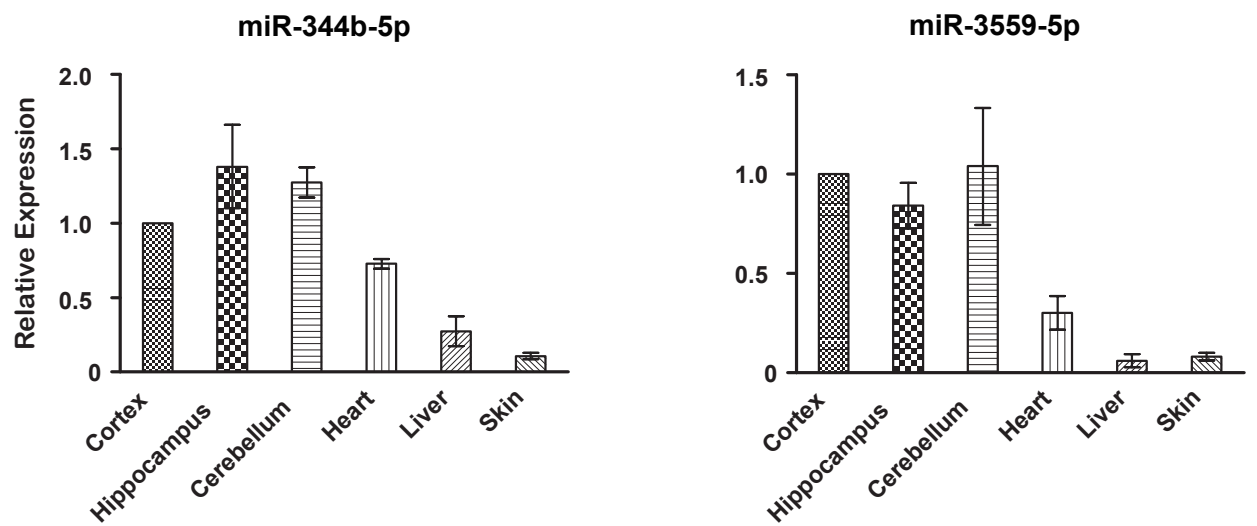

Supplement: Additional file 3 Figure S3. — Validation of the expression of miR-344b-5p and miR-3559-5p. The expression of miR-344b-2 gradually elevated during development. Expression of miR-3559-3p dropped over development, with a peak at E13. These two miRNAs highly enriched in central never system. [file 1471-2164-13-232-S3.pdf]

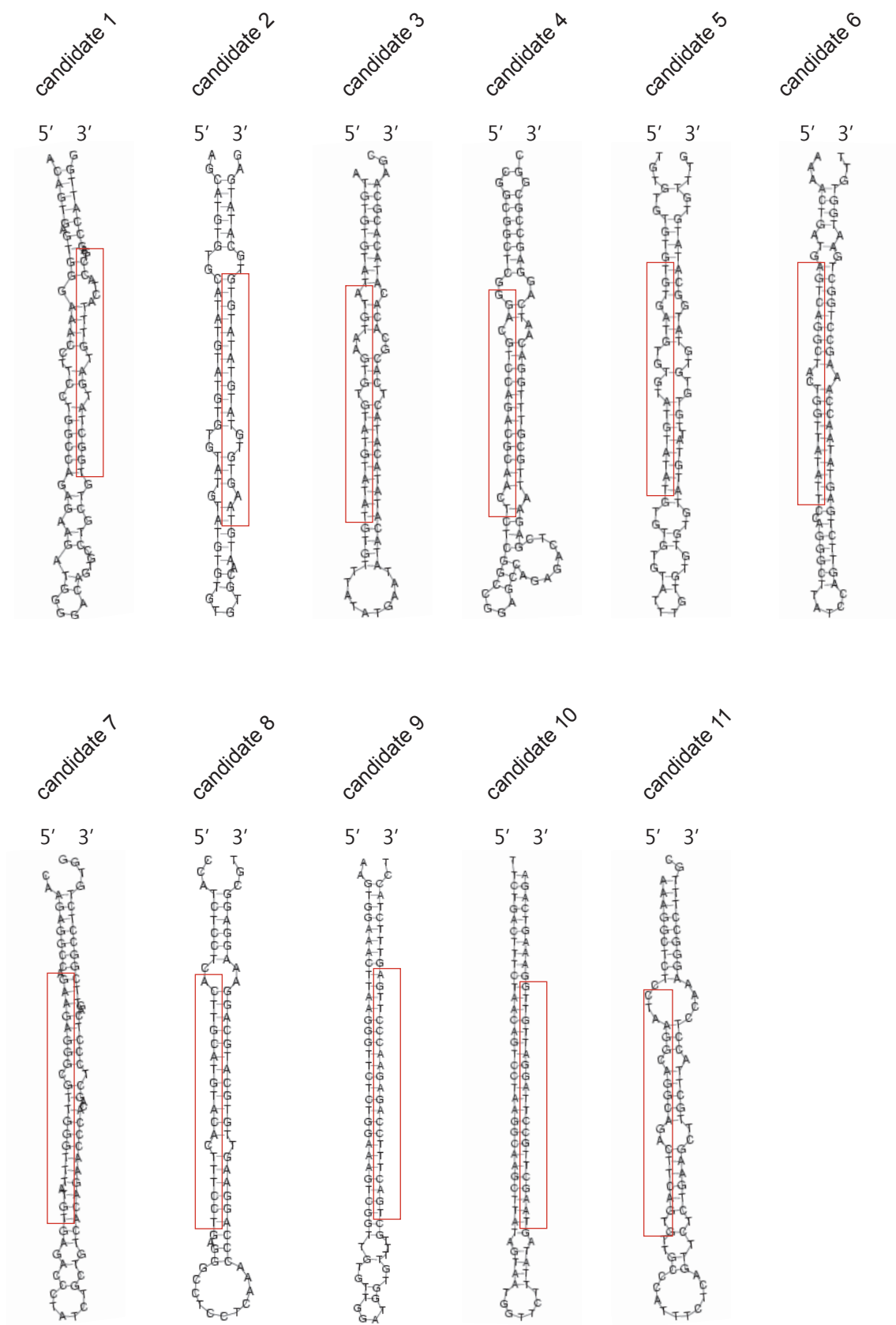

Supplement: Additional file 4 Figure S4. — Predicted structures of newly identified miRNAs. Computationally predicted secondary structures of the primary miRNA transcripts of 11 selected novel miRNA candidates are shown. Mature miRNA sequences are shown in the red frame. [file 1471-2164-13-232-S4.pdf]

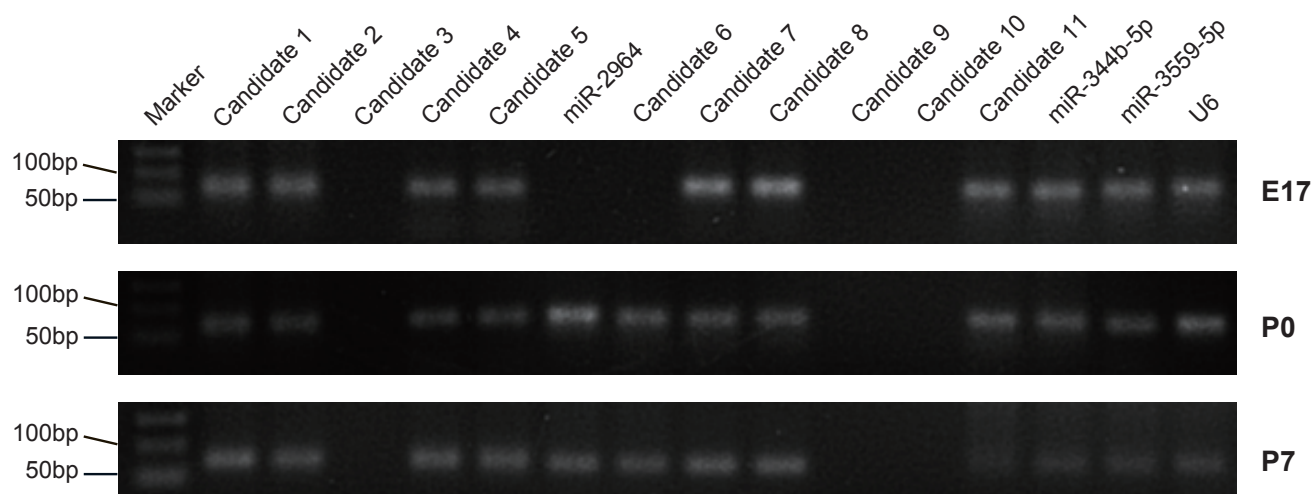

Yao et. al; Fig. S5

Supplement: Additional file 5 Figure S5. — PCR detection of novel miRNA candidates. Three known miRNAs, miR-2964, miR-344b-5p, and miR-3559-5p were also included as control. [file 1471-2164-13-232-S5.pdf]

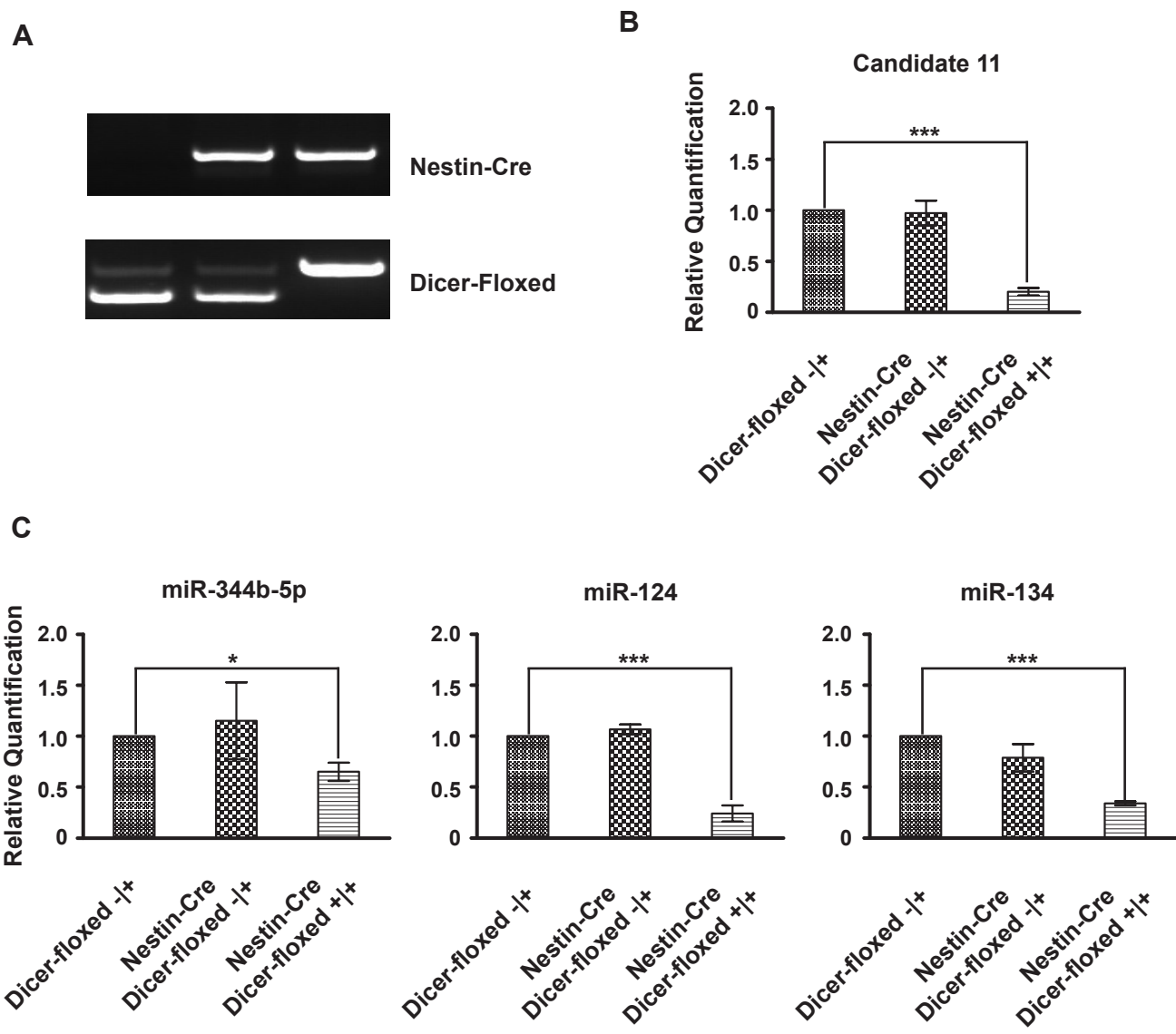

Supplement: Additional file 6 Figure S6. — Detection of mouse homologue of Candidate 11 in cortical tissue of mutant mouse with brain-specific knockout of Dicer. A. Genotyping of mutant mice. Nestin-Cre allele generated one band. Heterozygous Dicer-floxed allele generated two bands and homozygous allele generated one upper band. B. Expression level of novel Candidate 11 in P0 cortical tissue of Dicer knockout (Nestin-cre/Dicer-floxed+/+) mice revealed by qPCR. Expression level of Candidate 11 significantly decreased in knockout mice. C. Expression level of three known miRNAs, miR-344b-3p, miR-124, and miR-134, in P0 cortical tissue of wide type and Dicer knockout mice revealed by qPCR. Expression level of the three known miRNAs was remarkably decreased in knockout mice. [file 1471-2164-13-232-S6.pdf]
