# Supplementary material for: Transcriptome analysis of microRNAs in developing cerebral cortex of rat
Source: BMC Genomics. 2012 Jun 12;13:232. doi: 10.1186/1471-2164-13-232 (PMC3441217; doi:10.1186/1471-2164-13-232)
Supplement: Additional file 7 Figure S7. — Summary of potential isomiRs in cortical tissues. A. Summary of the fraction of each major group of isomiR in cortical tissue. Variability in the length of miRNAs was detected as addition and/or trimming of nucleotides at either 3’ end or 5’ end of mature miRNAs. Note that the 3’ end trimming is the most frequent type of modification in all samples. B. A summary of the sequence, length, and count of each isomiR of miR-128 in P14 cortical tissues. [file 1471-2164-13-232-S7.pdf]

A

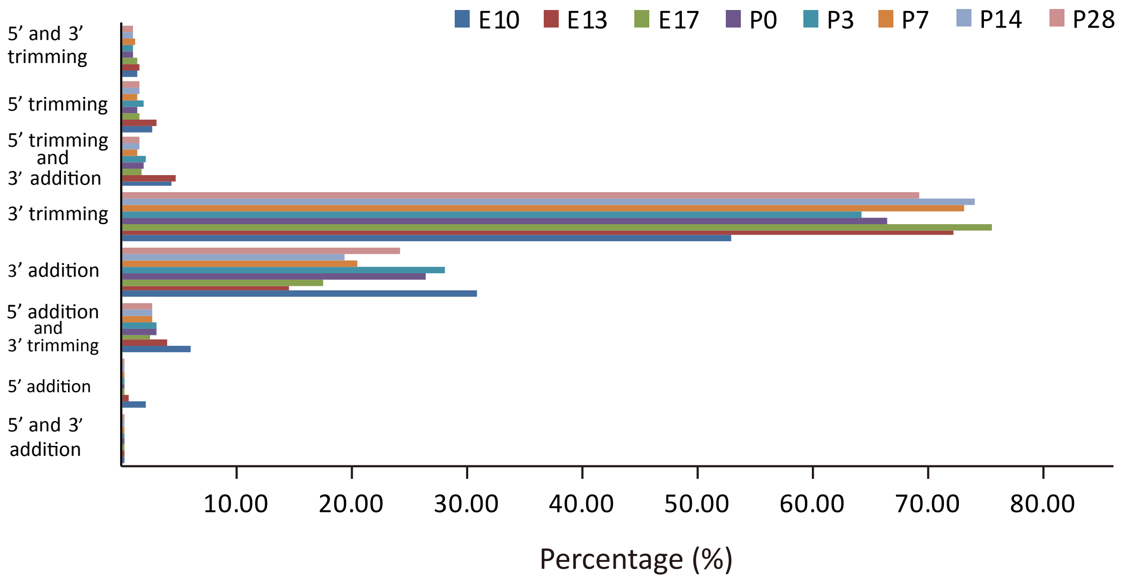

B

Type of isomiR	Sequence	Length	Counts
pre-miR-128	ATTTCTCACAGTGAACCGGTCTCTTTTTCAG		
miR-128	..........TCACAGTGAACCGGTCTCTTT..........	21	771473
5′addition and 3′addition	........TCTCACAGTGAACCGGTCTCTTTT.........	24	8
	.........CTCACAGTGAACCGGTCTCTTTT.........	23	428
	.........CTCACAGTGAACCGGTCTCTTTTT........	24	28
5′addition	.........CTCACAGTGAACCGGTCTCTTT..........	22	968
	........TCTCACAGTGAACCGGTCTCTTT..........	23	17
5′addition and 3′trimming	........TCTCACAGTGAACCGGTCTC............	20	3
	........TCTCACAGTGAACCGGTCTCT...........	21	2
	........TCTCACAGTGAACCGGTC..............	18	1
	.........CTCACAGTGAACCGGTCTCTT..........	21	132
	.........CTCACAGTGAACCGGTCTCT...........	20	101
	.........CTCACAGTGAACCGGTCTC............	19	100
	.........CTCACAGTGAACCGGTCT.............	18	75
5′trimming	............ACAGTGAACCGGTCTCTTT..........	19	8684
	...........CACAGTGAACCGGTCTCTTT..........	20	3091
	.............CAGTGAACCGGTCTCTTT..........	18	25
5′trimming and 3′addition	...........CACAGTGAACCGGTCTCTTTT.........	21	1465
	............ACAGTGAACCGGTCTCTTTT.........	20	4260
	...........CACAGTGAACCGGTCTCTTTTT........	22	221
	............ACAGTGAACCGGTCTCTTTTT........	21	282
	............ACAGTGAACCGGTCTCTTTTTC.......	22	4
	..............CAGTGAACCGGTCTCTTTT........	19	12
	..............CAGTGAACCGGTCTCTTTTT.......	20	1
	...............AGTGAACCGGTCTCTTTT........	18	16
	...............AGTGAACCGGTCTCTTTTTCA.....	21	4
5′ and 3′ trimming	.............AGTGAACCGGTCTCTTTTT.........	19	2
	.............ACAGTGAACCGGTCTCTT..........	18	1577
	............CACAGTGAACCGGTCTCT...........	18	446
	............CACAGTGAACCGGTCTCTT..........	19	472
3′ addition	...........TCACAGTGAACCGGTCTCTTTT........	22	355613
	...........TCACAGTGAACCGGTCTCTTTTT.......	23	21175
	...........TCACAGTGAACCGGTCTCTTTTTC......	24	45
	...........TCACAGTGAACCGGTCTCTTTTTCA.....	25	2
3′ trimming	...........TCACAGTGAACCGGTCTCT...........	19	100584
	...........TCACAGTGAACCGGTCTCTT..........	20	115538
	...........TCACAGTGAACCGGTCTC............	18	72211

Yao et.al;Fig. S7
