# Supplementary material for: Transcriptome analysis of microRNAs in developing cerebral cortex of rat
Source: BMC Genomics. 2012 Jun 12;13:232. doi: 10.1186/1471-2164-13-232 (PMC3441217; doi:10.1186/1471-2164-13-232)
Supplement: Additional file 8 Figure S8. — Detail editing profile of miR-128 during cortical development. A summary of the position, sequence, abundance (TPM) of each detected editing of miR-128 is shown. The high-abundance edited positions are highlighted with red color. [file 1471-2164-13-232-S8.pdf]

Edited position	sequence	E10	E13	E17	P0	P3	P7	P14	P28
**Wild type**	**TCACAGTG**	**1454**	**4553**	**20503**	**56260**	**100878**	**143098**	**164443**	**135771**
C / A ; 2	TAACAGTG	0.00	0.00	8.00	15.63	4.26	82.31	55.74	58.31
C / T ; 2	TTACAGTG	0.00	0.00	9.09	24.44	23.47	91.15	62.88	66.85
C / G ; 2	TGACAGTG	0.00	0.00	0.00	0.00	4.89	30.58	0.00	15.13
A / T ; 3	TCTCAGTG	0.00	0.00	4.49	9.02	9.44	73.03	58.64	34.85
A / C ; 3	TCCCAGTG	0.00	0.00	3.41	19.54	36.45	105.04	466.40	24.87
A / G ; 3	TCGCAGTG	0.00	0.00	6.30	17.96	26.52	95.35	80.67	74.28
C / A ; 4	TCAAAGTG	0.00	0.00	2.16	11.05	3.74	25.45	18.08	111.82
C / T ; 4	TCATAGTG	0.00	3.14	15.23	45.97	52.63	134.13	93.91	306.37
C / G ; 4	TCAGAGTG	0.00	0.00	0.00	16.59	5.13	22.66	20.46	202.16
A / T ; 5	TCACTGTG	0.00	0.00	2.54	8.79	4.05	37.87	77.04	38.30
A / C ; 5	TCACCGTG	0.00	0.00	0.00	8.32	25.89	12.45	370.24	13.16
A / G ; 5	TCACGGTG	0.00	0.00	6.54	13.80	14.85	57.78	95.27	57.96
G / A ; 6	TCACAATG	0.00	0.00	5.14	14.72	13.37	68.97	107.54	67.14
G / T ; 6	TCACATTG	2.62	0.00	17.04	75.82	88.45	349.73	645.63	126.26
G / C ; 6	TCACACTG	0.00	0.00	0.00	5.71	11.50	38.18	193.27	19.81
T / A ; 7	TCACAGAG	0.00	0.00	12.09	24.16	15.08	88.63	125.20	77.27
T / C ; 7	TCACAGCG	0.00	3.30	12.26	24.67	35.93	79.91	178.76	81.30
T / G ; 7	TCACAGGG	0.00	0.00	6.35	24.83	17.91	81.30	120.25	23.36
G / A ; 8	TCACAGTA	0.00	0.00	8.86	25.86	52.38	73.14	77.01	91.50
G / T ; 8	TCACAGTT	0.00	4.89	78.96	148.27	500.88	175.12	191.03	292.27
G / C ; 8	TCACAGTC	0.00	0.00	7.22	28.37	113.11	30.30	313.34	38.19
**Wild type**	**CGGTCTCTT**	**1454**	**4553**	**20503**	**56260**	**100878**	**143098**	**164443**	**135771**
C / A ;11	AGGTCTCTT	0.00	0.00	2.33	3.90	2.42	0.00	0.00	0.00
C / G ;11	GGGTCTCTT	0.00	0.00	0.00	6.06	15.83	0.00	12.06	0.00
T / G ;17	CGGTCGCTT	0.00	0.00	0.65	0.00	0.00	0.00	0.00	0.00
C / A ;18	CGGTCTATT	0.00	0.00	1.51	3.66	2.19	0.00	0.00	0.00
C / G ;18	CGGTCTGTT	0.00	0.00	1.67	3.76	0.00	0.00	0.00	0.00
C / T ;18	CGGTCTCTT	0.00	1.40	0.00	0.00	0.00	0.00	0.00	0.00
T / C ;19	CGGTCTCTT	0.00	0.00	0.00	1.29	2.38	2.25	2.35	2.28

Yao et.al;Fig. S8
