# Supplementary material for: Transcriptome analysis of microRNAs in developing cerebral cortex of rat
Source: BMC Genomics. 2012 Jun 12;13:232. doi: 10.1186/1471-2164-13-232 (PMC3441217; doi:10.1186/1471-2164-13-232)
Supplement: Additional file 9 Table S1. — Summary of reads from the deep-sequencing results. [file 1471-2164-13-232-S9.pdf]

Table S1: Summary of reads from deep-sequencing of small RNAs

	E10	E13	E17	P0	P0 ′	P3	P7	P14	P28
**Total reads**	23255034	23136784	20419875	20403949	19361274	21853490	19539424	22437354	20678150
**Clean reads**	16420636	12769251	13830486	14858820	13964298	13129806	14607976	15806174	16590379
**Mapped to genome**	12034079	10820400	11019566	11732561	10188829	11624211	12973582	13234099	10457484
